# Annual acknowledgement of reviewers

**DOI:** 10.1186/s12918-015-0150-6

**Published:** 2015-02-19

**Authors:** Tim Sands

**Affiliations:** BioMed Central, Floor 6, 236 Gray’s Inn Road, London, WC1X 8HB, UK

## Abstract

The editors of BMC Systems Biology would like to thank all our reviewers who have contributed to the journal in Volume 8 (2014).

**Reza Ahmadian**

Germany

**Réka Albert**

USA

**Joan Albiol**

Spain

**Antonio Alonso**

Spain

**Gökmen Altay**

Turkey

**Elena Alvarez**-**Buylla**

Mexico

**Rui Alves**

Spain

**Bharath Ananthasubramaniam**

Germany

**Erick Antezana**

Belgium

**Maciek Antoniewicz**

USA

**Yazdan Asgari**

Iran

**Scott Auerbach**

USA

**Simon Avery**

United Kingdom

**Ferhat Ay**

USA

**Joel Bader**

USA

**Shweta Bagewadi**

Germany

**Raffaella Balestrini**

Italy

**Christopher Banerji**

United Kingdom

**Julio Banga**

Spain

**Eudes Barbosa**

Denmark

**Chris Barnes**

United Kingdom

**Gregory Batt**

France

**David Baumler**

USA

**Joel Beaudouin**

Germany

**Vincenzo Belcastro**

Italy

**Christian Bender**

Germany

**Matthew Bennett**

USA

**Samuel Bernard**

France

**Mike Betenbaugh**

USA

**Kaustubh Bhalerao**

USA

**Gyan Bhanot**

USA

**Hans Binder**

Germany

**Michael Blinov**

USA

**Alexander Bockmayr**

Germany

**Laszlo Boros**

Hungary

**Luca Bortolussi**

Italy

**Daniel Braas**

USA

**Jay Brenman**

USA

**Gordon Broderick**

Canada

**Eric Bullinger**

Germany

**Helen Byrne**

United Kingdom

**Laurence Calzone**

France

**Ross Carlson**

USA

**Luis Caspeta**

Mexico

**Michele Ceccarelli**

Italy

**Gunnar Cedersund**

Sweden

**Sutirtha Chakraborty**

USA

**Tingfung Chan**

Hong Kong

**Nagasuma Chandra**

India

**Sriram Chandrasekaran**

USA

**Matthew Wook Chang**

Singapore

**Claudine Chaouiya**

Portugal

**Daniel Charlier**

Belgium

**Bor**-**Sen Chen**

Taiwan

**Chun Chen**

USA

**Tao Chen**

China

**Jin Chen**

USA

**Linyi Chen**

Taiwan

**Xing Chen**

China

**Sehyo Charley Choe**

Germany

**Lily Chylek**

USA

**Manfred Claassen**

Switzerland

**Diana Clausznitzer**

Germany

**Melissa Cline**

USA

**Carsten Conradi**

Germany

**Travis Craddock**

USA

**Péter Csermely**

Hungary

**Qinghua Cui**

China

**Tobias Czauderna**

Australia

**Ouiza Dahmoune**

Canada

**Luis De Figueiredo**

United Kingdom

**Gabriel Del Rio**

Mexico

**Peter D**’**Eustachio**

USA

**Julie Dickerson**

USA

**Elena Dimitrova**

USA

**Jürgen Dohmen**

Germany

**Mirela Domijan**

United Kingdom

**Francis Doyle**

USA

**Andreas Dräger**

USA

**Marine Dumousseau**

United Kingdom

**Steffen Durinck**

USA

**Maxime Durot**

France

**Daniel Dwyer**

USA

**Oliver Ebenhoeh**

Germany

**Birgitta Ebert**

Germany

**James Eddy**

USA

**Olivier Elemento**

USA

**Frank Emmert**-**Streib**

United Kingdom

**James Faeder**

USA

**François Fages**

France

**Xiaohui Fan**

China

**Howard Federoff**

USA

**Adam Feist**

USA

**Elisenda Feliu**

Denmark

**Sarah**-**Maria Fendt**

Belgium

**Xueyang Feng**

USA

**Jerome Feret**

France

**Fulvia Ferrazzi**

Germany

**Eugénio Ferreira**

Portugal

**Andres Florez**

Colombia

**Stephen Fong**

USA

**Christian Forst**

USA

**Elisa Franco**

USA

**Holger Fröhlich**

Germany

**Tobias Fuhrer**

Switzerland

**Andre Fujita**

Brazil

**Akira Funahashi**

Japan

**Jianxi Gao**

USA

**Ronald Germain**

USA

**James Gomes**

India

**Dominic Gruen**

Germany

**Zhenglong Gu**

USA

**Kunkun Guo**

China

**Shailendra Gupta**

Germany

**Jing**-**Dong Han**

China

**Benjamin Heavner**

USA

**Tomas Helikar**

USA

**Esteban Hernandez Vargas**

Germany

**Karsten Hiller**

Luxembourg

**Stefan Hohmann**

Sweden

**Christian Hong**

USA

**Raquel Hontecillas**

USA

**Pingzhao Hu**

Canada

**Valerie Hu**

USA

**Hsuan**-**Cheng Huang**

Taiwan

**Tao Huang**

China

**Michael Hucka**

USA

**Marc**-**Thorsten Hütt**

Germany

**Wilhelm Huisinga**

Germany

**Liliana Ironi**

Italy

**Johannes Jaeger**

Spain

**Mohit Jain**

USA

**Sarath Chandra Janga**

USA

**Laura Jarboe**

USA

**Lars Juhl Jensen**

Denmark

**Guangxu Jin**

USA

**Yufang Jin**

USA

**Iain Johnston**

United Kingdom

**Ozan Kahramanogullari**

Italy

**Christoph Kaleta**

Germany

**Guy Karlebach**

Germany

**Peter Karp**

USA

**Michael Katze**

USA

**Kathryn Kavanagh**

USA

**Benjamin Kellman**

USA

**Ekta Khurana**

USA

**Andrzej Kierzek**

United Kingdom

**Kyoung Heon Kim**

South Korea

**Jae Kyoung Kim**

USA

**Philip Kim**

Canada

**Yasuhiro Kobayashi**

Japan

**Mark Kon**

USA

**Hidetoshi Konno**

Japan

**Tamas Korcsmaros**

Hungary

**Frank Kramer**

Germany

**Robert Küffner**

Germany

**Inna Kuperstein**

France

**Zoltan Kutalik**

Switzerland

**Xin Lai**

Germany

**Kenneth Langlands**

United Kingdom

**Inna Lavrik**

Germany

**Alan Yueh**-**Luen Lee**

Taiwan

**Dong**-**Yup Lee**

Singapore

**Sang Yup Lee**

South Korea

**Jonathan Lees**

United Kingdom

**Jinzhi Lei**

China

**Forian Leitner**

Spain

**Ney Lemke**

Brazil

**Johan Leveau**

USA

**Jennifer Levering**

USA

**Jeffrey Lewis**

USA

**Jun Li**

USA

**Lei Li**

Canada

**Li Li**

Germany

**Jun Li**

USA

**Peng Li**

USA

**Yue Li**

Canada

**Jie Liang**

USA

**Jörg Linde**

Germany

**Michal Linial**

Israel

**Fen Liu**

China

**Jian Liu**

USA

**Yu**-**Fan Liu**

Taiwan

**Yang**-**Yu Liu**

USA

**James Lu**

Switzerland

**Timo Lubitz**

Germany

**Augustin Luna**

USA

**Feng Luo**

USA

**Xiaoyu Luo**

United Kingdom

**Aldons Lusis**

USA

**Hongwu Ma**

United Kingdom

**Huanfei Ma**

China

**Ben Macarthur**

United Kingdom

**Aidan Macnamara**

United Kingdom

**Paul Magwene**

USA

**Radhakrishnan Mahadevan**

Canada

**Ryszard Maleszka**

Australia

**Ashutosh Malhotra**

Germany

**Madhav Marathe**

USA

**Gaston Kuzamunu Mazandu**

South Africa

**Tommaso Mazza**

Italy

**Martin Meier**-**Schellersheim**

USA

**Hans Meinhardt**

Germany

**Pedro Mendes**

United Kingdom

**Luis Mendoza**

Mexico

**Eduardo Mendoza**

Germany

**Thomas Merritt**

Canada

**Christian Metallo**

USA

**Tom Michoel**

United Kingdom

**Gerald Miller**

USA

**Kathryn Miller**-**Jensen**

USA

**Gary Mirams**

United Kingdom

**Vladimir Mironov**

Norway

**Hugh Mitchell**

USA

**Pedro Monteiro**

Portugal

**Gabriel Moreno**-**Hagelsieb**

Canada

**Ivan Mura**

Colombia

**David Murrugarra**

USA

**Atul Narang**

India

**Satyaprakash Nayak**

USA

**Lan Nguyen**

Ireland

**David Nickerson**

New Zealand

**Qing Nie**

USA

**Jens Nielsen**

Sweden

**Florian Nigsch**

USA

**Emrah Nikerel**

Turkey

**Kang Ning**

China

**Daniel Noguera**

USA

**Victor Norris**

France

**Chris Oates**

United Kingdom

**Cihan Oguz**

USA

**Guilherme Oliveira**

Brazil

**Brett Olivier**

Netherlands

**Zoltan Oltvai**

USA

**Irene Otero Muras**

Spain

**Alberto Paccanaro**

United Kingdom

**Ranadip Pal**

USA

**Di Pan**

USA

**Linqiang Pan**

China

**Michael Pargett**

USA

**Joel Parker**

USA

**Kiran Raosaheb Patil**

Germany

**Josch Pauling**

Denmark

**Linda Petzold**

USA

**Alex Phipps**

United Kingdom

**Alexander Pico**

USA

**Jesus Pico**

Spain

**Esa Pitkänen**

Finland

**Francisco Planes**

Spain

**Chris Pritchett**

USA

**Michel Puceat**

France

**Xiaoning Qian**

USA

**Lake**-**Ee Quek**

Australia

**Nicole Erika Radde**

Germany

**Prahlad Ram**

USA

**Doraiswami Ramkrishna**

USA

**Elisabeth Remy**

France

**Andrea Ribeiro**

Portugal

**Fabio Rinaldi**

Switzerland

**Zweigerdt Robert**

Germany

**Gene Robinson**

USA

**Marcus Roper**

USA

**Paul Ruet**

France

**Derek Ruths**

Canada

**David Safranek**

Czech Republic

**Leonor Saiz**

USA

**Uwe Sauer**

Switzerland

**Jörg Schaber**

Germany

**Sascha Schäuble**

Germany

**Nico Scherf**

Germany

**Falk Schreiber**

Germany

**Charles Schroeder**

USA

**Stefan Schuster**

Germany

**Jean**-**Marc Schwartz**

United Kingdom

**Thomas Schwarzl**

Ireland

**Timothy Secomb**

USA

**Nicola Segata**

Italy

**Daniel Segrè**

USA

**Sylvain Sené**

France

**Yao Shen**

USA

**Hiroshi Shimizu**

Japan

**Fumihide Shiraishi**

Japan

**Kenneth Siddle**

United Kingdom

**Heike Siebert**

Germany

**Anne Siegel**

France

**Vangelis Simeonidis**

Luxembourg

**Age Smilde**

Netherlands

**Lucian Smith**

USA

**Michael Sokolov**

Switzerland

**Mingzhou Song**

USA

**Linsheng Song**

China

**Michael Springer**

USA

**Friedrich Srienc**

USA

**Natalie Stanford**

United Kingdom

**Dov Stekel**

United Kingdom

**Angelique Stephanou**

France

**Adam Stevens**

United Kingdom

**Gautier Stoll**

France

**Adam Streck**

Germany

**Mark Styczynski**

USA

**Neil Swainston**

United Kingdom

**William Swindell**

USA

**Takako Takai**-**Igarashi**

Japan

**Yinjie Tang**

USA

**Andreas Tauch**

Germany

**Charles Taylor**

USA

**Andrew Teschendorff**

China

**Nathalie Théret**

France

**Reuben Thomas**

USA

**Xiaojun Tian**

USA

**Hendrik Tiedemann**

Germany

**Tomas Tokar**

Canada

**Laurent Tournier**

France

**Cong Trinh**

USA

**Yufeng Jane Tseng**

Taiwan

**Peter Uetz**

USA

**Rajanikanth Vadigepalli**

USA

**Jan Bert Van Klinken**

Netherlands

**Natal Van Riel**

Netherlands

**Fabio Vandin**

Denmark

**Alan Veliz**-**Cuba**

USA

**Pasquale Verde**

Italy

**Jean**-**Philippe Vert**

France

**Alejandro Fernández Villaverde**

Spain

**Arunachalam Vinayagam**

USA

**Dawn Walker**

United Kingdom

**Dagmar Waltemath**

Germany

**Dirk Walther**

Germany

**Feng**-**Sheng Wang**

Taiwan

**Edwin Wang**

Canada

**Jin Wang**

USA

**Jing Wang**

USA

**Rui**-**Sheng Wang**

USA

**Tao Wang**

USA

**Xinkun Wang**

USA

**Jochen Weile**

Canada

**Thomas Werner**

Germany

**Ann West**

USA

**Margaret Westfall**

USA

**Tyisha Williams**

USA

**Sarala Wimalaratne**

United Kingdom

**Carsten Wiuf**

Denmark

**Katy Wolstencroft**

Netherlands

**Troy Wood**

USA

**Ming Wu**

USA

**Xuebing Wu**

USA

**Zhi Xie**

USA

**Yu Xue**

China

**Jinn**-**Moon Yang**

Taiwan

**Xin**-**She Yang**

United Kingdom

**Xuerui Yang**

China

**Levent Yilmaz**

USA

**Shanye Yin**

USA

**Nir Yosef**

USA

**Naif Zaman**

Canada

**Jürgen Zanghellini**

Austria

**Hossein Zare**

USA

**Sven Zenker**

Germany

**Jinghui Zhang**

USA

**Qiang Zhang**

USA

**Weijia Zhang**

USA

**Wenxiao Zhao**

China

**Yuqi Zhao**

USA

**Du Zhou**

China

